# Simulated dynamics of southern cattle fever ticks (*Rhipicephalus* (*Boophilus*) *microplus*) in south Texas, USA: investigating potential wildlife-mediated impacts on eradication efforts

**DOI:** 10.1186/s13071-021-04724-3

**Published:** 2021-05-01

**Authors:** Hsiao-Hsuan Wang, William E. Grant, Pete D. Teel, Kimberly H. Lohmeyer, Adalberto A. Pérez de León

**Affiliations:** 1 Ecological Systems Laboratory, Department of Ecology and Conservation Biology, Texas A&M University, College Station, TX 77843 USA; 2 Department of Entomology, Texas A&M AgriLife Research, College Station, TX 77843 USA; 3 Knipling-Bushland U.S. Livestock Insects Research Laboratory and Veterinary Pest Genomics Center, United States Department of Agriculture – Agricultural Research Service, Kerrville, TX 78028 USA; 4 San Joaquin Valley Agricultural Sciences Center, United States Department of Agriculture – Agricultural Research Service, Parlier, CA 93648 USA

**Keywords:** Agent-based model, Cattle Fever Tick Eradication Program, Host community, Individual-based model, Integrated tick management, Nilgai, White-tailed deer

## Abstract

**Background:**

Cattle fever ticks (CFT), *Rhipicephalus* (*Boophilus*) *annulatus* and *R*. (*B*.) *microplus*, are vectors of microbes causing bovine babesiosis and pose a threat to the economic viability of the US livestock industry. Efforts by the Cattle Fever Tick Eradication Program (CFTEP) along the US-Mexico border in south Texas are complicated by the involvement of alternate hosts, including white-tailed deer (*Odocoileus virginianus*) and nilgai (*Boselaphus tragocamelus*).

**Methods:**

In the present study, we use a spatially explicit, individual-based model to explore the potential effects of host species composition and host habitat use patterns on southern cattle fever ticks (SCFT, *R*. (*B*.) *microplus*) infestation dynamics and efficacy of eradication schemes.

**Results:**

In simulations without eradication efforts, mean off-host larval densities were much higher when cattle were present than when only white-tailed deer and nilgai were present. Densities in mesquite and meadows were slightly higher, and densities in mixed brush were much lower, than landscape-level densities in each of these scenarios. In eradication simulations, reductions in mean off-host larval densities at the landscape level were much smaller when acaricide was applied to cattle only, or to cattle and white-tailed deer, than when applied to cattle and nilgai. Relative density reductions in mesquite, mixed brush, and meadows depended on host habitat use preferences. Shifting nilgai habitat use preferences increasingly toward mixed brush and away from mesquite did not change mean off-host larval tick densities noticeably at the landscape level. However, mean densities were increased markedly in mesquite and decreased markedly in mixed brush, while no noticeable change in density was observed in meadows.

**Conclusions:**

Our results suggest that continued integration of field data into spatially explicit, individual-based models will facilitate the development of novel eradication strategies and will allow near-real-time infestation forecasts as an aid in anticipating and preventing wildlife-mediated impacts on SCFT eradication efforts.
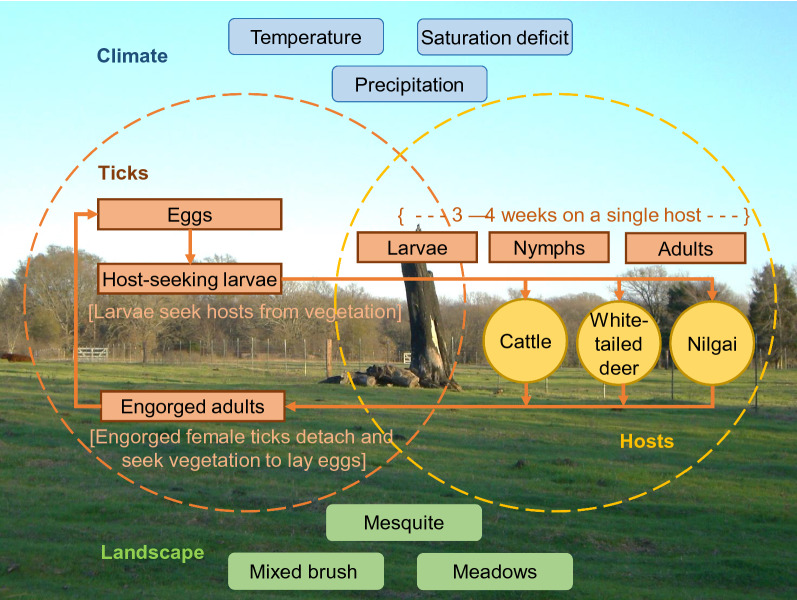

**Supplementary Information:**

The online version contains supplementary material available at 10.1186/s13071-021-04724-3.

## Background

Cattle fever ticks (CFT), *Rhipicephalus* (*Boophilus*) *annulatus* and *R*. (*B*.) *microplus*, were eradicated from the USA in 1943 through efforts of the Cattle Fever Tick Eradication Program (CFTEP); however, they have again become a threat to the economic viability of the US livestock industry [1]. In addition to the direct effects they cause on livestock health as ectoparasites, CFT are also the vectors of *Babesia bigemina* and *B. bovis*, which cause bovine babesiosis, and *Anaplasma marginale*, which causes anaplasmosis in cattle [2, 3]. The CFTEP maintains a permanent quarantine zone in south Texas along the Rio Grande to buffer CFT incursions from Mexico, where the tick vectors and bovine babesiosis remain endemic [4, 5].

Historically, operations by the CFTEP focused on monitoring cattle, the primary host of CFT. However, the presence of increasing numbers of alternative host species in the border region poses significant challenges to the CFTEP [1, 6]. White-tailed deer (*Odocoileus virginianus*) maintain CFT populations in the absence of cattle, which compromises eradication efforts [7, 8]. Nilgai (*Boselaphus tragocamelus*) is also an alternate host of CFT in the south Texas-Mexico transboundary region [9, 10]. Nilgai is an antelope species in the same Bovidae family as cattle, which was introduced to south Texas from India in 1941 [11].

CFT outbreaks have increased recently in Texas outside the quarantine zone (in Cameron, Hidalgo, Jim Wells, Jim Hogg, and Willacy counties; 15 May 2020 notice from the Texas Animal Health Commission) [12]. Continued success of the CFTEP requires an integrated strategy based on an interdisciplinary systems approach that specifically includes consideration of management risks and opportunities associated with the livestock–wildlife interface; simulation modeling has been identified as a useful tool in this regard [13, 14]. There is a wide variety of CFT models focused on questions dealing with both basic biology and management on four continents [15]. However, relatively few models have included explicit representation of wildlife hosts [16–19], and only one has included nilgai [20]. Based on results of a simulation modeling study focused on CFT–livestock–wildlife interactions, it has been suggested that refugia for CFT could be created by white-tailed deer during acaricide treatments directed at cattle via deposition and subsequent collection of CFT in habitats favorable for survival of off-host life stages [18]. Further simulation studies have suggested that nilgai, which roam widely over parts of south Texas and northeastern Mexico [21], could augment the role of white-tailed deer in sustaining CFT infestations by facilitating widespread redistribution of CFT from refugia [20]. Nilgai activity ranges are larger than those of white-tailed deer by almost an order of magnitude; the maximum axis of home ranges of radio-tracked nilgai have exceeded 30 km [11]. Male nilgai are capable of traversing such distances in a single day [22].

One of the major uncertainties in dealing with potential wildlife-mediated impacts on CFT eradication efforts remains our lack of knowledge about the combined effects of host species composition and host habitat use patterns on tick infestation dynamics. Wang et al. [20] conducted a preliminary evaluation of the sensitivities of southern cattle fever tick (SCFT, *R*. (*B*.) *microplus*) dynamics to simulated changes in each of these three factors, individually, in the absence of eradication efforts. These authors found, not surprisingly, that SCFT dynamics in general, and off-host larval dynamics in particular, were sensitive to changes in each factor. These authors also simulated various infestation and eradication scenarios, using literature-based estimates of each of these three factors. In all of their simulated infestation scenarios, the proportion of infested landscape patches was highest in poor tick habitat and lowest in fair tick habitat. They defined “good” (mesquite-dominated woody plant communities), “fair” (mixed thorn shrub communities), and “poor” (uncanopied forage areas) in terms of the relative survival rates of off-host larvae. These counterintuitive results were the product of the species-specific habitat use patterns of hosts combined with the habitat-specific survival rates of off-host larvae. When these authors simulated eradication scenarios, the particular host species that received acaricide treatments determined the proportions of landscape patches in the different tick habitats that remained infested.

In the present study, we explore in greater depth the effects of host species composition and host habitat use patterns on tick infestation dynamics and efficacy of eradication schemes. More specifically, we simulate scenarios in which acaricide is applied to cattle only and scenarios in which acaricides are also applied to one or both of the two principal wildlife hosts (white-tailed deer and nilgai). We evaluate the efficacy of each acaricide application scheme in terms of the resulting densities of off-host tick larvae. We also attempt to distinguish the influence of host habitat use preferences from that of host species composition per se, and further explore the potential importance of nilgai habitat use patterns. Thus the main purpose of this modeling exercise is explanation, sensu [23]. We use a model containing explicit causal pathways relating host habitat use preferences and host species composition to off-host larval densities, which generates output supportive results of field studies documenting involvement of white-tailed deer and nilgai as SCFT hosts [6, 7, 21, 24], to explain how wildlife hosts could impact tick eradication efforts.

## Methods

**Fig. 1:**
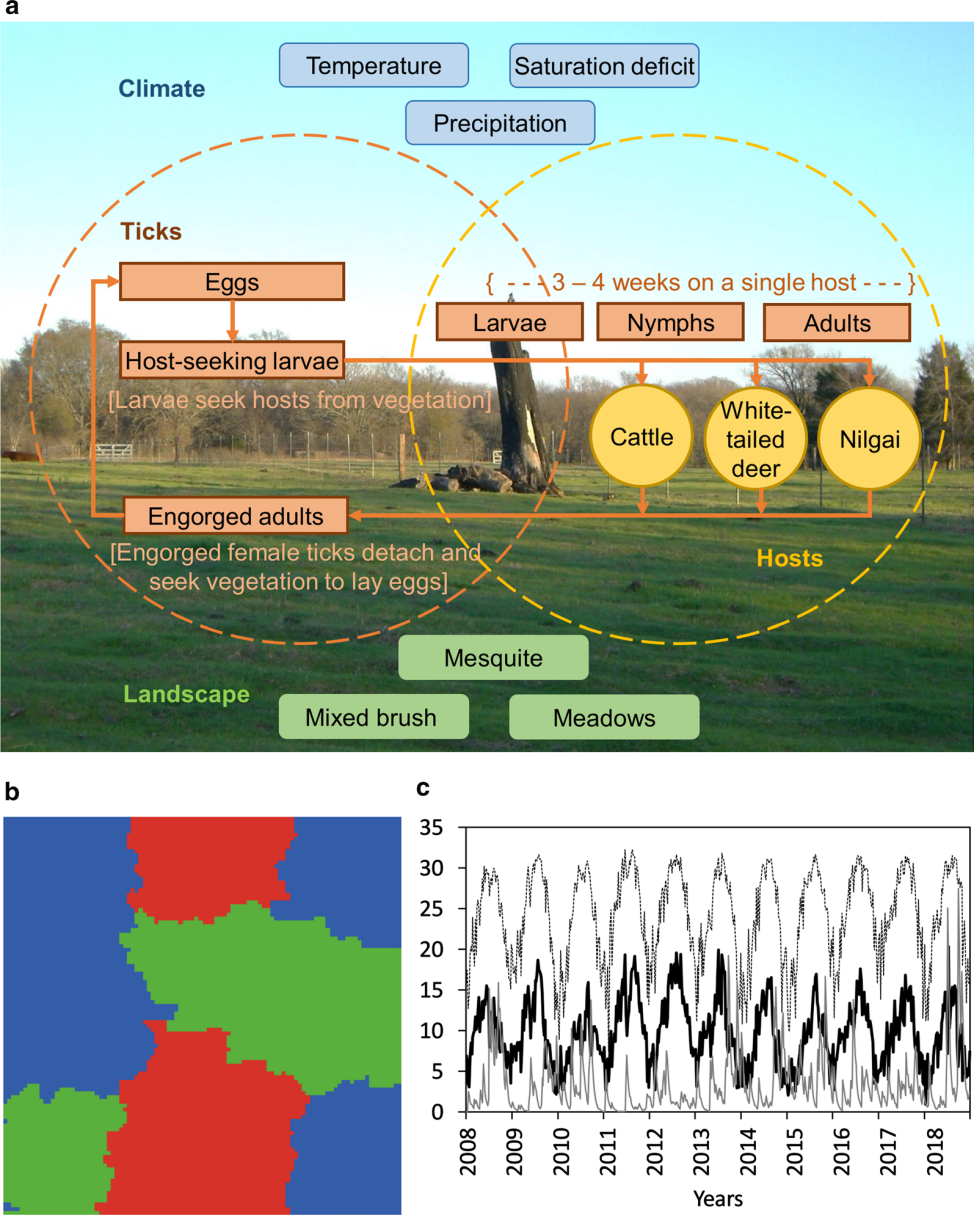
Conceptual model of the system of interest (**a**) modified from Fig. 1 in [18] and Fig. 1 in [20]. Landscape configuration (**b**) consisting of 31% mesquite (green), 28% mixed brush (red), and 41% meadows (blue). Weekly air temperatures (C, black dashed line), saturation deficits (millibars, bold black line), and index values based on precipitation (cm, gray line) (**c**) recorded in south Texas (Willacy County), USA from January 2008 through December 2018 (Climate Data Online, National Oceanic and Atmospheric Administration, https://www.ncdc.noaa.gov/cdo-web/)

To investigate potential causal mechanisms responsible for wildlife-mediated impacts on SCFT eradication efforts, we used the model described by Wang et al. [18, 20]. The model is spatially explicit and individual-based [25, 26], and is designed to simulate effects of shifts in the spatiotemporal patterns of host (cattle, white-tailed deer, and nilgai) habitat use on the dynamics of SCFT populations (Fig. 1a). The model is parameterized to represent a hypothetical 10,000-hectare ranch comprising 31% mesquite-dominated, 28% mixed-brush-dominated, and 41% open meadow (uncanopied grass) habitats (equivalent to relatively good, fair, and poor habitats, respectively, for survival of off-host tick larvae) (Fig. 1b) under weather conditions recorded in south Texas (Willacy County), USA from January 2008 through December 2018 (Climate Data Online, National Oceanic and Atmospheric Administration, https://www.ncdc.noaa.gov/cdo-web/) (Fig. 1c). A detailed description of the basic model following the standard ODD (overview, design concepts, and details) protocol for describing individual-/agent-based models [25, 26] is available in Wang et al. [18, 20]; a description of the major processes represented in the model can be found in Additional file 1, and a list of parameter values for the baseline version of the model used in the present study appears in Table 1.

**Table 1:** The parameters used to represent nilgai, cattle, and white-tailed deer as hosts of cattle fever ticks, their baseline values, and their information sources

Parameters	Values	References
Cattle/ha	0.0286	[52]
Deer/ha	0.1667	[52]
Nilgai/ha	0.05	[53]
Activity area (ha) cattle	300	[18]
Activity area (ha) deer	675	[54]
Activity area (ha) nilgai	8355	[11]
Hab pref mesquite^a^ cattle	0.30	[52]
Hab pref mixed brush^b^ cattle	0.10	[52]
Hab pref meadows^c^ cattle	0.60	[52]
Hab pref mesquite^a^ deer	0.20	[52]
Hab pref mixed brush^b^ deer	0.40	[52]
Hab pref meadows^c^ deer	0.40	[52]
Hab pref mesquite^a^ nilgai	0.30	[53]
Hab pref mixed brush^b^ nilgai	0.10	[53]
Hab pref meadows^c^ nilgai	0.60	[53]

^a^“Mesquite” refers to mesquite-dominated woody plant community and is considered a relatively “good” climatic environment for CFT to complete the off-host portion of the life cycle and sustain larval survival

^b^“Mixed brush” refers to a community of mixed thorn shrub species and is considered a relatively “fair” climatic environment for CFT to complete the off-host portion of the life cycle and sustain larval survival

^c^“Meadow” refers to uncanopied forage areas and is considered a relatively “poor” climatic environment for CFT to complete the off-host portion of the life cycle and sustain larval survival

Our experimental design consisted of simulating SCFT dynamics on the hypothetical ranch over a 5-year period under each of seven scenarios. Three scenarios involved different combinations of host species in which no eradication schemes were applied. Four scenarios involved different eradication schemes in which all three host species were present (Table 2). Host species composition in the three scenarios without eradication schemes included: Scenario (1) cattle, white-tailed deer, and nilgai; Scenario (2) only cattle; and Scenario (3) only white-tailed deer and nilgai. Eradication schemes in the four scenarios with all three host species present included acaricide treatments applied to: Scenario (4) cattle only; Scenario (5) cattle and white-tailed deer only; Scenario (6) cattle and nilgai only; and Scenario (7) all three host species. We assumed that acaricide applied to cattle killed 100% of on-host ticks, and acaricide applied to white-tailed deer and nilgai killed 100% of on-host ticks on 50% of the individuals, 50% of the ticks on 25% of the individuals, and failed to kill any ticks on 25% of the individuals. The variable efficacy by host type reflects the difficulties of treating wildlife for tick suppression [27]. For each of these scenarios, we ran 10 replicate stochastic (Monte Carlo) simulations because the variation among 10 simulations showed no statistical difference based on Friedman tests. Each simulation was initiated with no off-host ticks and one half the maximum tick load on one individual of the non-acaricide-treated host species. During each simulation, beginning in year 2, we monitored off-host larval tick densities in each landscape cell and summarized these results in terms of densities at the landscape level, and in mesquite, mixed-brush, and meadow habitats.

**Table 2:** Summary of the experimental design for simulations, which consisted of seven scenarios

Scenario number	Species present	Species treated
1	Cattle, white-tailed deer, and nilgai	None
2	Cattle	None
3	White-tailed deer and nilgai	None
4	Cattle, white-tailed deer, and nilgai	Cattle
5	Cattle, white-tailed deer, and nilgai	Cattle and white-tailed deer
6	Cattle, white-tailed deer, and nilgai	Cattle and nilgai
7	Cattle, white-tailed deer, and nilgai	Cattle, white-tailed deer, and nilgai

Three scenarios involved different combinations of host species in which no eradication schemes were applied. Four scenarios involved different eradication schemes in which all three host species were present

To separate the influence on our results of host habitat use preferences from that of host species composition per se, we also repeated the experimental design assuming no host habitat use preference. That is, we replaced the preferences used by Wang et al. [18] with habitat use preferences equal to relative habitat availability, thus distributing host use approximately uniformly (host movements are probabilistic) across the landscape. A *t* test was used to compare off-host larval tick densities from each scenario simulated with, versus without, host habitat use preferences. Statistical analyses were performed using R 3.4.4 (R Core Team, 2013) and under the *α* = 0.01 significance threshold.

Finally, based on the results of the experiments above, to further explore the relative importance of nilgai habitat use preferences on off-host larval tick densities, we repeated the eradication schemes (Scenarios 4 through 6 only) for each of three nilgai habitat use preferences. We increased nilgai preference for mixed brush (fair SCFT habitat) from 0.1 to 0.4 in increments of 0.1, while simultaneously reducing preference for mesquite (good SCFT habitat) from 0.3 to 0 in increments of 0.1. Habitat use preferences for cattle and white-tailed deer were those used by Wang et al. [18].

## Results

**Fig. 2:**
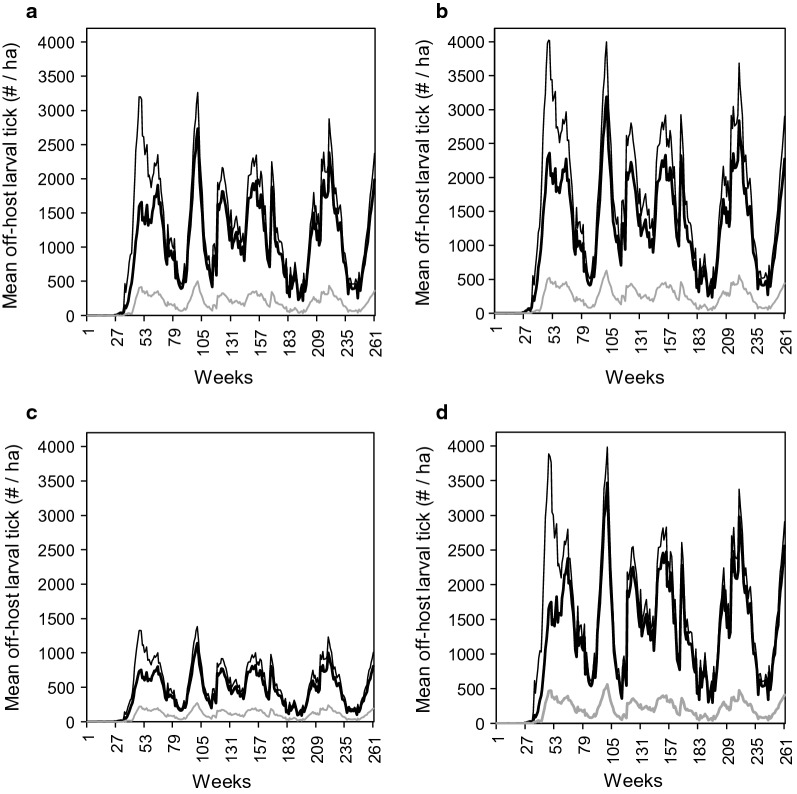
Temporal trends in mean off-host larval tick densities (per ha) (**a**) at the landscape level, (**b**) in mesquite, (**c**) in mixed brush, and (**d**) in meadows from scenarios with cattle, white-tailed deer, and nilgai (Scenario 1, black lines), only cattle (Scenario 2, thick black lines), and only white-tailed deer and nilgai (Scenario 3, gray lines). No eradication schemes were applied in these scenarios

When no eradication schemes were applied, at the landscape level there was relatively little difference in temporal trends in mean off-host larval tick densities between the two scenarios in which cattle were present (Scenario 1 and Scenario 2), but during simulations of the scenario in which only white-tailed deer and nilgai were present (Scenario 3), densities were much lower (Fig. 2a). Relative densities among scenarios in mesquite (Fig. 2b), mixed brush (Fig. 2c), and meadows (Fig. 2d) followed the same temporal pattern exhibited at the landscape level (Fig. 2a), with densities higher with cattle than without cattle. Relative densities among habitat types were similar within each of the three scenarios, with densities in mesquite (Fig. 2b) and meadows (Fig. 2d) slightly higher than densities at the landscape level (Fig. 2a), and densities in mixed brush (Fig. 2c) much lower. Weather-imposed seasonal and year-to-year fluctuations in densities were the same in all simulations.

**Fig. 3:**
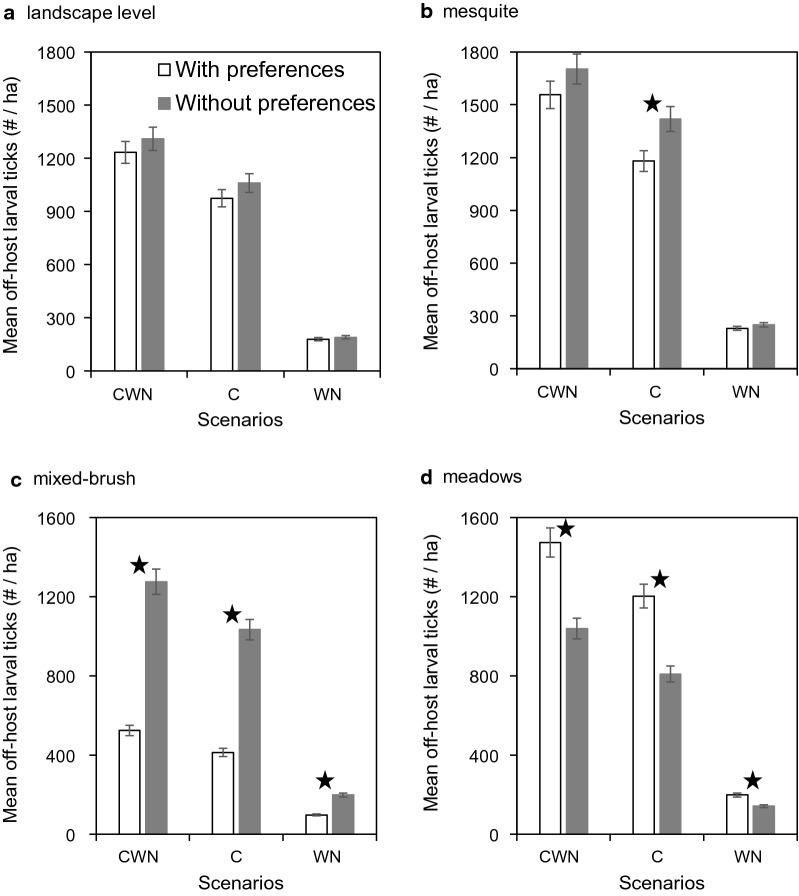
Comparison of host habitat use preference on mean off-host larval tick densities (per ha) (± SE) (**a**) at the landscape level, (**b**) in mesquite, (**c**) in mixed brush and (**d**) in meadows from scenarios with cattle, white-tailed deer, and nilgai (Scenario 1, CWN), only cattle (Scenario 2, C), and only white-tailed deer and nilgai (Scenario 3, WN). Results are shown for simulations with (white bars) and without (gray bars) host habitat use preferences. Star symbol indicates that the difference is statistically significant at *p* ≤ 0.01. No eradication schemes were applied in these scenarios

In the no-eradication scenarios (Scenario 1, Scenario 2, and Scenario 3), when we compared off-host larval tick densities from each scenario simulated with, versus without, host habitat use preferences, there were no differences (CWN: *t* = –1.4630, *df* = 18, *p* = 0.1607; C: *t* = –2.0270, *df* = 18, *p* = 0.0577; WN: *t* = 1.411, *df* = 18, *p* = 0.1752) at the landscape level (Fig. 3a) or almost no difference (CWN: *t* = –2.0200, *df* = 18, *p* = 0.0585; C: *t* = –4.4420, *df* = 17, *p* = 0.0004; WN: *t* = –19.410, *df* = 18, *p* = 0.0681) in mesquite (Fig. 3b). However, in the absence of host habitat use preferences, mean densities were more than twice as high (CWN: *t* = –18.7000, *df* = 12, *p* < 0.0001; C: *t* = –19.1100, *df* = 12, *p* < 0.0001; WN: *t* = –15.6800, *df* = 13, *p* < 0.0001) in mixed brush (Fig. 3c) and statistically lower (CWN: *t* = 8.2668, *df* = 16, *p* < 0.0001; C: *t* = 9.3246, *df* = 16, *p* < 0.0001; WN: *t* = 7.9531, *df* = 16, *p* < 0.0001) in meadows (Fig. 3d).

**Fig. 4:**
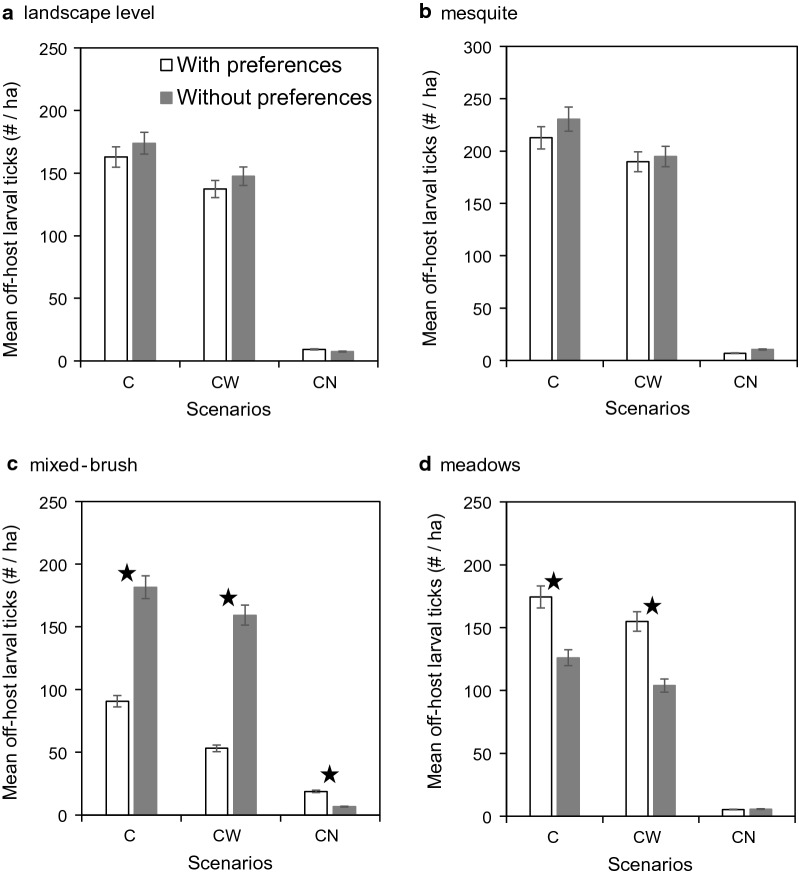
Comparison of host habitat use preference on mean off-host larval tick densities (per ha) (± SE) (**a**) at the landscape level, (**b**) in mesquite, (**c**) in mixed brush, and (**d**) in meadows from scenarios in which acaricides were applied to cattle only (Scenario 4, C), cattle and white-tailed deer only (Scenario 5, CW), and cattle and nilgai only (Scenario 6, CN). Results are shown for simulations with (white bars) and without (gray bars) host habitat use preferences. Star symbol indicates that the difference is statistically significant at *p* ≤ 0.01. All three host species were present in these scenarios (results for Scenario 7 not shown; highest mean off-host larval tick densities < 1/ha when acaricides were applied to all three host species)

When eradication schemes were applied, reductions in mean off-host larval tick densities at the landscape level were much smaller when acaricide was applied to cattle only (Scenario 4) or to cattle and white-tailed deer only (Scenario 5) than when applied to cattle and nilgai only (Scenario 6) (Fig. 4a) (results for Scenario 7 not shown in Fig. 4; highest mean off-host larval tick densities were < 1/ha when acaricides were applied to all three host species). Relative density reductions in mesquite (Fig. 4b), mixed brush (Fig. 4c), and meadows (Fig. 4d) followed the same general pattern across eradication schemes exhibited at the landscape level (Fig. 4a). However, reductions across eradication schemes in mixed brush were significantly larger (CWN: *t* = –15.5100, *df* = 13, *p* < 0.0001; C: *t* = –21.6900, *df* = 11, *p* < 0.0001; WN: *t* = 20.8920, *df* = 11, *p* < 0.0001) in simulations without, than in simulations with, host habitat use preferences (Fig. 4c), whereas most reductions in meadows were significantly larger (CWN: *t* = 7.6974, *df* = 16, *p* < 0.0001; C: *t* = 9.3726, *df* = 16, *p* < 0.0001; WN: *t* = –1.5140, *df* = 18, *p* = 0.1474) in simulations with host preferences (Fig. 4d).

**Fig. 5:**
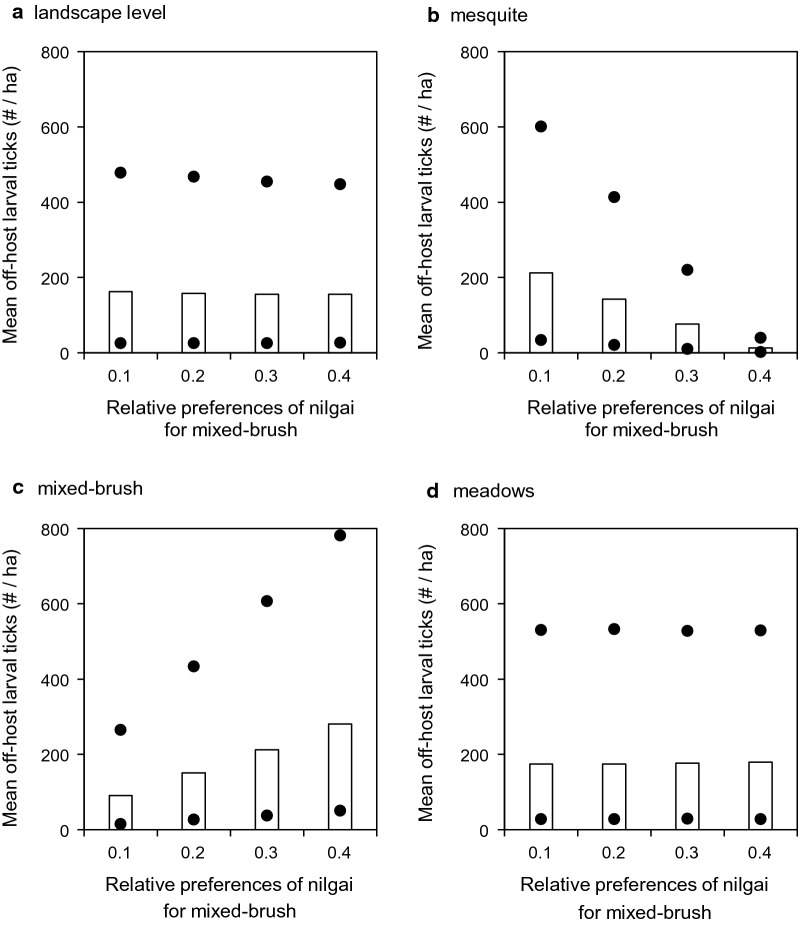
Effects of altering nilgai habitat use preferences on mean off-host larval tick densities (per ha) (**a**) at the landscape level, (**b**) in mesquite, (**c**) in mixed brush, and (**d**) in meadows when acaricides were applied to cattle only (Scenario 4). All three host species were present in this scenario. Bars and dots represent mean, maximum, and minimum densities that occurred during years 2 through 5 of simulations

**Fig. 6:**
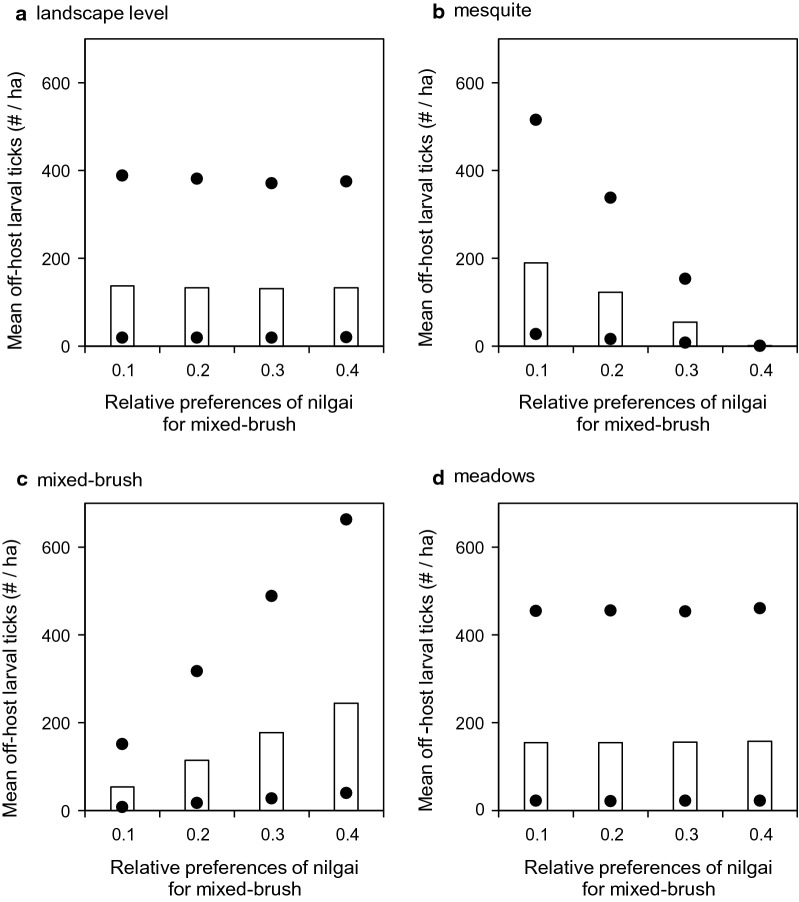
Effects of altering nilgai habitat use preferences on mean off-host larval tick densities (per ha) (**a**) at the landscape level, (**b**) in mesquite, (**c**) in mixed brush, and (**d**) in meadows when acaricides were applied to cattle and white-tailed deer only (Scenario 5). All three host species were present in this scenario. Bars and dots represent mean, maximum, and minimum densities that occurred during years 2 through 5 of simulations

**Fig. 7:**
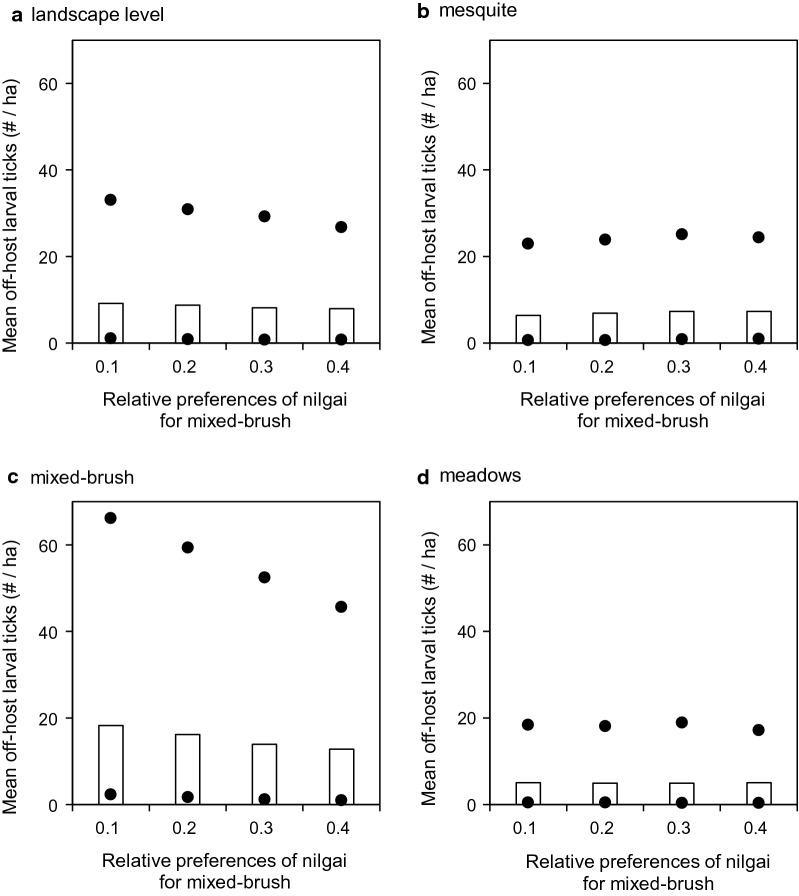
Effects of altering nilgai habitat use preferences on mean off-host larval tick densities (per ha) (**a**) at the landscape level, (**b**) in mesquite, (**c**) in mixed brush, and (**d**) in meadows when acaricides were applied to cattle and nilgai only (Scenario 6). All three host species were present in this scenario. Bars and dots represent mean, maximum, and minimum densities that occurred during years 2 through 5 of simulations

Shifting nilgai habitat use preferences increasingly toward mixed brush and away from mesquite in the simulations when acaricide treatments were applied to cattle only (Scenario 4) did not change mean off-host larval tick densities noticeably at the landscape level (Fig. 5a). However, mean densities were increased in mesquite by ≈94% (from ≈13 to ≈213 larvae/ha, Fig. 5b) and decreased in mixed brush by ≈308% (from ≈280 to ≈91 larvae/ha, Fig. 5c), while densities in meadows were not changed noticeably (Fig. 5d). When acaricide treatments were applied to cattle and white-tailed deer only (Scenario 5), results were similar qualitatively. Densities were relatively unchanged at the landscape level (Fig. 6a) and in meadows (Fig. 6d), but increased in mesquite by > 99% (from < 1 to ≈190 larvae/ha, Fig. 6b) and decreased in mixed brush by ≈460% (from ≈244 to ≈53 larvae/ha, Fig. 6c). When acaricide treatments were applied to cattle and nilgai only (Scenario 6), results again were similar qualitatively, but all mean densities were approximately an order of magnitude lower than the corresponding densities in the other two scenarios (Fig. 7).

## Discussion

Simulations presented here, which were generated by a model containing explicit causal pathways relating host habitat use preferences and host species composition to off-host larval tick densities, provided numerical output supporting field studies documenting involvement of white-tailed deer and nilgai as SCFT hosts [6, 7, 21, 24]. That is, our model represents a plausible set of cause–effect relationships that provide an explanation of the manner in which white-tailed deer and nilgai could be involved as SCFT hosts. Thus, by logical extension, our model represents a plausible set of cause–effect relationships that provide an explanation of the manner in which white-tailed deer and nilgai could impact tick eradication efforts. Such explanation informs the exploration of new tactics for controlling the spread of CFT by wildlife hosts, as well as the development of novel strategies for sustainable CFT eradication in general, which are urgently needed [27–29].

The simulated dynamics of our model indicate that eradication efforts are challenged not only by the presence of wildlife hosts per se, but by our uncertainty regarding their habitat use patterns [30]. Although potential effects of species composition and relative abundance of wildlife hosts on system-level densities of host-seeking larvae are easily explained, wildlife-mediated effects on distribution and abundance of larvae among different habitats remain to be fully understood [31–33]. Thus, our simulations provide insight into the potential impact of wildlife host habitat preferences on distribution patterns of host-seeking larvae. For example, host-seeking larval densities were relatively higher in meadows than in mixed brush in our simulations using a set of literature-based host habitat use preferences (Fig. 3). However, in simulations assuming no host habitat use preferences, larval densities were markedly higher in mixed brush than in meadows. These results can be understood considering higher survival rates of off-host tick life stages in mixed brush, which would result in higher densities given the equal deposition of engorged females when host habitat preferences are completely absent.

Host-seeking larval distribution patterns may be sensitive to subtler shifts in wildlife host habitat preferences. When we shifted nilgai preferences for mixed brush from 0.1 toward 0.4 (and simultaneously preferences for mesquite from 0.3 toward 0), host-seeking larval densities in the most infested landscape cells increased approximately threefold in mixed brush and decreased by approximately one-third in mesquite in those scenarios (4 and 5) in which nilgai were not treated with acaricide (Figs. 5, 6). These results suggest the potential for creation of host-seeking larval “hotspots,” which might effectively become larval refugia during acaricide-based eradication schemes. Wang et al. [18] reported that white-tailed deer might facilitate the development of such refugia. Preliminary results suggested that nilgai might serve to widely redistribute ticks from these refugia [20]. However, the aforementioned studies did not explicitly examine nilgai habitat use. Agudelo et al. [19] simulated effects on larval distribution based on shifts of white-tailed deer habitat preferences in more detail and found that densities were highest in mesquite if the time spent in mesquite/mixed brush was roughly double that assumed by Wang et al. [18]. However, if time spent in mesquite was lower (≤ 0.1), host-seeking larvae were essentially confined within mixed-brush refugia.

Information on the ecology of refugia for CFT larvae in south Texas originally posited through modeling of the livestock–wildlife interface is emerging [18, 34]. Further, the larval hotspots revealed by our expanded model, which also simulates the effect of nilgai, present both challenges and opportunities with regard to the development of new tactics for controlling SCFT larvae on, or deposited by, wildlife hosts [15]. This challenge can be met by integrating knowledge of wildlife movements, CFT biology, and management strategies from the perspective of rangelands as complex adaptive systems [30, 35, 36]. Habitat use preferences of white-tailed deer have been studied extensively [37–42], but seldom within the context of their effects on CFT management strategies [21, 22]. Nilgai movements have been studied within the context of CFT management, with an interest in their ability to spread CFT widely because of their potentially large home ranges (571–29,909 ha) [21], and diurnal activity patterns of nilgai have been studied with an interest in increasing efficacy of CFT treatment schemes [43]. However, information on nilgai use of habitat shared with white-tailed deer and their preferred movement patterns in and around the CFTEP quarantine zone in south Texas remains scarce [44–46]. Furthermore, the landscape mosaic within which nilgai, as well as white-tailed deer and other potential wildlife hosts, form their habitat use patterns is swiftly changing due to rural land fragmentation [47].

## Conclusions

New tactics for controlling the spread of CFT by wildlife hosts, as well as developing novel strategies for sustainable CFT eradication in general, are urgently needed [27–29]. The principle of “agri-intelligence” can be applied to identify the interactions of environmental and anthropogenic factors that generate the temporal and spatial patterns of CFT outbreaks in south Texas. In this context, it is critical to base an integrated CFT management strategy on an interdisciplinary systems approach [48], which specifically considers risks and opportunities associated with the livestock–wildlife interface, and is informed by models that enable full-spectrum thinking for resilience of eradication efforts [13]. Existing technology allows the collection and integration of detailed data on animal movements, physical structure, and vegetative composition of the landscape as well as microclimatic conditions. We suggest that continual integration of such data into spatially explicit, individual-based models will facilitate the development of novel eradication strategies [49, 50] and will allow near-real-time infestation forecasts [51] as an aid in anticipating and preventing wildlife-mediated impacts on CFT eradication efforts.

## Supplementary Information

**Additional file 1**: Supplementary methods for “Simulated dynamics of southern cattle fever ticks (*Rhipicephalus* (*Boophilus*) *microplus*) in south Texas, USA: Investigating potential wildlife-mediated impacts on eradication efforts.

## Data Availability

The simulated data generated and/or analyzed during the present study are available from the corresponding author upon reasonable request.

## Abbreviations

CFT: Cattle fever tick
SCFT: Southern cattle fever tick
CFTEP: Cattle Fever Tick Eradication Program

## References

[1] Pérez de León AA, Strickman DA, Knowles DP, Fish D, Thacker E, de La Fuente J (2010). One Health approach to identify research needs in bovine and human babesioses: workshop report. Parasit Vectors.

[2] Pérez de León A, Vannier E, Alamazán C, Krause PJ, Sonenshine DE, Roe RM (2014). Tick-borne protozoa. Biology of ticks.

[3] Aubry P, Geale D (2011). A review of bovine anaplasmosis. Transbound Emerg Dis.

[4] Lohmeyer K, Pound J, May M, Kammlah D, Davey R (2011). Distribution of *Rhipicephalus* (*Boophilus*) *microplus* and *Rhipicephalus* (*Boophilus*) *annulatus* (Acari: Ixodidae) infestations detected in the United States along the Texas/Mexico border. J Med Entomol.

[5] Pérez de León AA, Teel PD, Li A, Ponnusamy L, Roe RM (2014). Advancing integrated tick management to mitigate burden of tick-borne diseases. Outlooks Pest Manage.

[6] Lohmeyer KH, May MA, Thomas DB, Pérez de León AA (2018). Implication of nilgai antelope (Artiodactyla: Bovidae) in reinfestations of *Rhipicephalus* (*Boophilus*) *microplus* (Acari: Ixodidae) in outh Texas: a review and update. J Med Entomol.

[7] Pound J, George J, Kammlah D, Lohmeyer K, Davey R (2010). Evidence for role of white-tailed deer (Artiodactyla: Cervidae) in epizootiology of cattle ticks and southern cattle ticks (Acari: Ixodidae) in reinfestations along the Texas/Mexico border in south Texas: a review and update. J Econ Entomol.

[8] Kistner TP, Hayes FA (1970). White-tailed deer as hosts of cattle fever-ticks. J Wildl Dis.

[9] Cárdenas-Canales EM, Ortega-Santos JA, Campbell TA, García-Vázquez Z, Cantú-Covarrubias A, Figueroa-Millán JV (2011). Nilgai antelope in northern Mexico as a possible carrier for cattle fever ticks and *Babesia bovis* and *Babesia bigemina*. J Wildl Dis.

[10] Goolsby JA, Singh NK, Ortega-S A, Hewitt DG, Campbell TA, Wester D (2017). Comparison of natural and artificial odor lures for nilgai (*Boselaphus tragocamelus*) and white-tailed deer (*Odocoileus virginianus*) in south Texas: developing treatment for cattle fever tick eradication. Int J Parasitol Parasit Wildl.

[11] Moczygemba JD, Hewitt DG, Campbell TA, Ortega-S JA, Feild J, Hellickson MW (2012). Home ranges of the nilgai antelope (*Boselaphus tragocamelus*) in Texas. Southwest Nat.

[12] TAHC. Notice to lvestock owners: Cattle fever ticks spreading in south Texas. Austin, TX: Texas Animal Health Commission; 2020.

[13] Pérez de León AA, Teel PD, Auclair AN, Messenger MT, Guerrero FD, Schuster G (2012). Integrated strategy for sustainable cattle fever tick eradication in USA is required to mitigate the impact of global change. Front Physiol.

[14] Wang H-H, Teel PD, Grant WE, Soltero F, Urdaz J, Ramírez AEP (2019). Simulation tools for assessment of tick suppression treatments of *Rhipicephalus* (*Boophilus*) *microplus* on non-lactating dairy cattle in Puerto Rico. Parasit Vectors.

[15] Wang HH, Corson MS, Grant WE, Teel PD (2017). Quantitative models of Rhipicephalus (Boophilus) ticks: historical review and synthesis. Ecosphere.

[16] Estrada-Peña A, Carreón D, Almazán C, de la Fuente J (2014). Modeling the impact of climate and landscape on the efficacy of white tailed deer vaccination for cattle tick control in northeastern Mexico. PLoS ONE.

[17] Zeman P, Lynen G (2010). Conditions for stable parapatric coexistence between Boophilusdecoloratus and *B. microplus* ticks: a simulation study using the competitive Lotka-Volterra model. Exp Appl Acarol.

[18] Wang H-H, Teel PD, Grant WE, Schuster G, Pérez de León A (2016). Simulated interactions of white-tailed deer (*Odocoileus virginianus*), climate variation and habitat heterogeneity on southern cattle tick (*Rhipicephalus* (*Boophilus*) *microplus*) eradication methods in south Texas, USA. Ecolog Model.

[19] Agudelo MS, Grant WE, Wang H-H (2021). Effects of white-tailed deer habitat use preferences on southern cattle fever tick eradication: simulating impact on pasture vacation strategies. Parasit Vectors.

[20] Wang H-H, Grant WE, Teel PD, Lohmeyer KH, Pérez de León AA (2020). Enhanced biosurveillance of high-consequence invasive pests: southern cattle fever ticks, *Rhipicephalus* (*Boophilus*) *microplus*, on livestock and wildlife. Parasit Vectors.

[21] Foley AM, Goolsby JA, Ortega-S A, Ortega-S JA, de León AP, Singh NK (2017). Movement patterns of nilgai antelope in south Texas: implications for cattle fever tick management. Prev Vet Med.

[22] Busch JD, Stone NE, Nottingham R, Araya-Anchetta A, Lewis J, Hochhalter C (2014). Widespread movement of invasive cattle fever ticks (*Rhipicephalus microplus*) in southern Texas leads to shared local infestations on cattle and deer. Parasit Vectors.

[23] Edmonds B, Le Page C, Bithell M, Chattoe-Brown E, Grimm V, Meyer R (2019). Different modelling purposes. J Artif Soc Soc Simul.

[24] Olafson PU, Thomas DB, May MA, Buckmeier BG, Duhaime RA (2018). Tick vector and disease pathogen surveillance of nilgai antelope (*Boselaphus tragocamelus*) in southeastern Texas, USA. J Wildl Dis.

[25] Grimm V, Berger U, Bastiansen F, Eliassen S, Ginot V, Giske J (2006). A standard protocol for describing individual-based and agent-based models. Ecol Model.

[26] Grimm V, Berger U, DeAngelis DL, Polhill JG, Giske J, Railsback SF (2010). The ODD protocol: a review and first update. Ecol Model.

[27] Currie CR, Hewitt DG, Ortega SJA, Schuster GL, Campbell TA, Lohmeyer KH (2020). Efficacy of white-tailed deer (*Odocoileus virginianus*) treatment for cattle fever ticks in southern Texas, USA. J Wildl Dis.

[28] Osbrink WLA, Showler AT, Abrigo V, Pérez de León AA (2020). *Rhipicephalus* (*Boophilus*) *microplus* (Ixodida: Ixodidae) larvae collected from vegetation in the coastal wildlife corridor of southern Texas and research solutions for integrated eradication. J Med Entomol.

[29] Thomas DB, Klafke G, Busch JD, Olafson PU, Miller RA, Mosqueda J (2020). Tracking the increase of acaricide resistance in an invasive population of cattle fever ticks (Acari: Ixodidae) and implementation of real-time PCR assays to rapidly genotype resistance mutations. Ann Entomol Soc Am.

[30] French JT, Wang H-H, Grant WE, Tomeček JM (2019). Dynamics of animal joint space use: a novel application of a time series approach. Move Ecol.

[31] Wang H-H, Grant W, Teel P (2012). Simulation of climate–host–parasite–landscape interactions: a spatially explicit model for ticks (Acari: Ixodidae). Ecol Model.

[32] Wang H-H, Grant WE, Teel PD, Hamer SA (2015). Simulation of climate-tick-host-landscape interactions: effects of shifts in the seasonality of host population fluctuations on tick densities. J Vector Ecol.

[33] Wang H-H, Grant WE, Teel PD, Hamer SA (2016). Tick-borne infectious agents in nature: simulated effects of changes in host density on spatial-temporal prevalence of infected ticks. Ecol Model.

[34] Showler AT, Pérez de León A (2020). Landscape ecology of *Rhipicephalus* (*Boophilus*) *microplus* (Ixodida: Ixodidae) outbreaks in the south Texas coastal plain wildlife corridor including man-made barriers. Environ Entomol.

[35] Wang H-H, Grant WE, Teague R (2020). Modeling rangelands as spatially-explicit complex adaptive systems. J Environ Manage.

[36] Teague R, Grant B, Wang H-H (2015). Assessing optimal configurations of multi-paddock grazing strategies in tallgrass prairie using a simulation model. J Environ Manage.

[37] Sanders CL (1963). Habitat preferences of the white-tailed deer and several exotic ungulates in south Texas. Ecology.

[38] Inglis JM, Hood RE, Brown BA, DeYoung CA (1979). Home range of white-tailed deer in Texas coastal prairie brushland. J Mammal.

[39] Beier P, McCullough DR. Factors influencing white-tailed deer activity patterns and habitat use. Wildl Monogr. 1990:3–51.

[40] Coe PK, Johnson BK, Stewart KM, Kie JG. Spatial and temporal interactions of elk, mule deer, and cattle. In: Transactions of the 69th North American Wildlife and Natural Resources Conference: March 16–20, 2004 2004; Spokane, WA: 656–69.

[41] Cooper SM, Perotto-Baldivieso HL, Owens MK, Meek MG, Figueroa-Pagan M (2008). Distribution and interaction of white-tailed deer and cattle in a semi-arid grazing system. Agr Ecosyst Environ.

[42] Brunjes KJ, Ballard WB, Humphrey MH, Harwell F, McIntyre NE, Krausman PR (2006). Habitat use by sympatric mule and white-tailed deer in Texas. J Wildl Manag.

[43] Singh NK, Miller RJ, Klafke GM, Goolsby JA, Thomas DB, de Leon AAP (2018). In-vitro efficacy of a botanical acaricide and its active ingredients against larvae of susceptible and acaricide-resistant strains of *Rhipicephalus* (*Boophilus*) *microplus* Canestrini (Acari: Ixodidae). Ticks Tick-borne Dis.

[44] Sheffield Jr WJ, Ables ED, Fall BA. Geographic and ecologic distribution of nilgai antelope in Texas. J Wildl Manage. 1971:250–7.

[45] Sheffield WJ, Fall BA, Brown BA (1983). The nilgai antelope in Texas.

[46] Mungall EC, Sheffield WJ (1994). Exotics on the range.

[47] Kjelland ME, Kreuter UP, Clendenin GA, Wilkins RN, Wu XB, Afanador EG (2007). Factors related to spatial patterns of rural land fragmentation in Texas. Environ Manage.

[48] Iwanaga T, Wang H-H, Hamilton SH, Grimm V, Koralewski TE, Salado A (2021). Socio-technical scales in socio-environmental modeling: managing a system-of-systems modeling approach. Environ Model Softw.

[49] Wang H-H, Grant WE, Elliott NC, Brewer MJ, Koralewski TE, Westbrook JK (2019). Integrated modelling of the life cycle and aeroecology of wind-borne pests in temporally-variable spatially-heterogeneous environment. Ecol Model.

[50] Koralewski TE, Wang H-H, Grant WE, Brewer MJ, Elliott NC, Westbrook JK (2020). Integrating models of atmospheric dispersion and crop-pest dynamics: linking detection of local aphid infestations to forecasts of region-wide invasion of cereal crops. Ann Entomol Soc Am.

[51] Koralewski TE, Wang H-H, Grant WE, LaForest JH, Brewer MJ, Elliott NC (2020). Toward near-real-time forecasts of airborne crop pests: aphid invasions of cereal grains in North America. Comput Electron Agric.

[52] Cooper SM, Perotto-Baldivieso HL, Owens MK, Meek MG, Figueroa-Pagán M (2008). Distribution and interaction of white-tailed deer and cattle in a semi-arid grazing system. Agric Ecosyst Environ.

[53] Sheffield WJ, Ables ED, Fall BA (1971). Geographic and ecologic distribution of nilgai antelope in Texas. J Wildl Manage.

[54] Hellickson MW, Campbell TA, Miller KV, Marchinton RL, DeYoung CA (2008). Seasonal ranges and site fidelity of adult male white-tailed deer (*Odocoileus virginianus*) in southern Texas. Southwest Nat.

