# Supplementary material for: Simulated dynamics of southern cattle fever ticks (Rhipicephalus (Boophilus) microplus) in south Texas, USA: investigating potential wildlife-mediated impacts on eradication efforts
Source: Parasit Vectors. 2021 May 1;14:231. doi: 10.1186/s13071-021-04724-3 (PMC8088722; doi:10.1186/s13071-021-04724-3)
Supplement: Supplementary file 1 — Additional file 1: Supplementary methods for “Simulated dynamics of southern cattle fever ticks (Rhipicephalus (Boophilus) microplus) in south Texas, USA: Investigating potential wildlife-mediated impacts on eradication efforts. [file 13071_2021_4724_MOESM1_ESM.pdf]

Additional file 1

Supplementary methods for "Simulated dynamics of southern cattle fever ticks (*Rhipicepalus* (*Boophilus*) *microplus*) in south Texas, USA: Investigating potential wildlife-mediated impacts on eradication efforts

The major processes represented in the various sub-models of the model developed by Wang et al. [1] are described below. In addition to white-tailed deer (*Odocoileus virginianus*), nilgai (*Boselaphus tragocamelus*) were included as a wildlife host in the model used by Wang et al. [2]. Major process described below for deer function in the same manner for nilgai. Parameters differentiating nilgai from white-tailed deer are presented in Table 1 of the main text.

Submodels

Execute tick control if applicable

At the beginning of each week, the program checks to see if tick control should be applied and, if so, executes the tick control method being simulated. Tick control methods include: (1) the application of acaricides to cattle every 2 weeks for 9 consecutive months, (2) the application of acaricides to cattle every 2 weeks for 12 consecutive months, and (3) the removal of cattle from a pasture ("pasture vacation") for 12 consecutive months. Assessment criteria include: (1) the relative density of host-seeking larvae in the environment, and the number of (2) adult ticks on cattle and (3) on deer prior to, during, and for several months following the application of each control method. The application of acaricides to cattle is represented by setting the values of the host attributes representing the number larval, nymphal, and adult ticks to zero. Pasture vacation is represented by temporarily removing cattle from the system. (In NetLogo, the individual hosts representing cattle "die" and new individuals representing cattle are "created" 12 months later.)

Update climatic conditions and recalculate associated off-host tick survival and development rates

At the beginning of each week, the program updates temperature, saturation deficit, and the precipitation index based on values in the corresponding time series of inputs (Section 2.6 in Wang et al. [1]). Based on these new environmental conditions, the program recalculates off-host survival and development rates for tick eggs, larvae, and engorged adults in each type of habitat, as well as the relative rate of host-seeking activity of off-host larvae, based on information presented by Mount et al. [3]. The program also calculates the number of eggs laid per ovipositing female as a function of temperature and the type of host from which the female obtained her blood meal based on information presented by Cooksey et al. [4]. The program calculates development rates of eggs and engorged (fed, after blood meal) adults as functions of cumulative "degree-weeks" of temperature; survival rates of eggs and off-host engorged adults as functions of current (weekly) temperature, saturation deficit, and precipitation, as modified by habitat type; and relative rates of host-seeking activity of off-host larvae (the proportion of off-host larvae that potentially could encounter and attach to a host) as functions of current (weekly) temperature following Mount et al. [3] (Appendix in Wang et al. [1]).

Update attributes of landscape cells representing number of ticks in each off-host developmental phase

At the beginning of each week, the program updates each landscape cell in terms of (1) the number of tick eggs, larvae, and engorged adults that have survived, (2) the number of eggs that have developed into larvae, (3) the number of engorged (female) adults that have laid eggs and died, and (4) the number of eggs laid. The survival of eggs, larvae, and engorged adults depends on temperature, saturation deficit, and the precipitation index, as well as the habitat type of the landscape cell following Mount et al. [3] (Appendix in Wang et al. [1]). The development of eggs and engorged adults depends on temperature following Mount et al. [3], and the number of eggs laid per engorged (female) adult depends on the species of the host from which the female obtained her blood meal following Cooksey et al. [4] (Appendix in Wang et al. [1]).

Calculate number of off-host larvae collected by hosts and update corresponding attributes of landscape cells and individual hosts

Thirty times during each week, preceding each of the 30 rounds of host movements (see Section "Move hosts within the landscape and update corresponding attributes of landscape cells and individual hosts" below), the program calculates the number of off-host larvae that are collected by each host from each landscape cell and updates the number of larvae in each cell and the number of larvae on each host. Each individual host has a probability of collecting larvae that depends upon the abundance and relative activity level of larvae in that cell, with an upper limit on the number of larvae that can be collected determined by the number of larvae already on the host. The relative activity level of off-host larvae depends on temperature following Mount et al. [3] and the maximum number of larvae allowed on a host depends on the species of the host (see Section 3 in Wang et al. [1]).

Calculate survival and development of on-host ticks, calculate number of on-host ticks deposited by hosts, and update corresponding attributes of landscape cells and individual hosts

Thirty times during each week, preceding each of the 30 rounds of host movements (see Section "Move hosts within the landscape and update corresponding attributes of landscape cells and individual hosts" below) and following the collection of off-host larvae during each round (see Section "Calculate number of off-host larvae collected by hosts and update corresponding attributes of landscape cells and individual hosts" above), the program calculates the survival and development of the larval, nymphal, and adult ticks on each host, and updates the corresponding attributes of each host. The duration of on-host life stages is one week for larvae, one week for nymphs, and one to two weeks for adults, following Mount et al. [3], and the on-host survival rates for larvae, nymphs, and adults also follow Mount et al. [3] (Appendix in Wang et al. [1]). The program then deposits 10% of the adult ticks that have been on a host for at least three weeks into the landscape cell in which the host currently is located and updates the corresponding attributes of the host and the landscape cell. Thus members of any given cohort of on-host adult ticks are deposited as engorged adults into up to 30 different landscape cells during the 30 host movements that occur during the last week that the adults are on the host.

Move hosts within the landscape and update corresponding attributes of landscape cells and individual hosts

Thirty times during each week, following the collection, on-host survival and development, and deposition of ticks during each of the 30 rounds, the program moves each host within the landscape. The landscape cells visited are selected probabilistically from those within the activity range of the host based on the habitat preferences of the host and the proportion of the different habitat types within the activity range. The program code that executes host movements is provided in the Appendix of Wang et al. [1]. Note that, since habitat cells within the simulated landscape are arrayed as a torus, the activity ranges of individuals are not limited by the edges of the 30 × 30 cell grid used for visual displays of the landscape. For example, if an individual's activity range is centered near the right-hand edge of the visual display grid, it "wraps around" onto the left-hand portion of the visual display grid. Thus, host movements are unconstrained by an "edge effect."

Write output summarizing landscape attributes, host attributes, and system-level statistics

At the end of each week, the program writes to text files summaries of climatic conditions (temperature, saturation deficit, precipitation index), selected attributes of landscape cells (density of off-host larvae), hosts (number of on-host adult ticks), and system-level statistics (mean density of off-host larvae in the system and in each habitat type, mean number of on-host adult ticks per individual for each host species).

References

1. Wang H-H, Teel PD, Grant WE, Schuster G, Pérez de León A. Simulated interactions of white-tailed deer (*Odocoileus virginianus*), climate variation and habitat heterogeneity on southern cattle tick (*Rhipicephalus* (*Boophilus*) *microplus*) eradication methods in south Texas, USA. Ecological Modelling. 2016;342:82-96.
2. Wang H-H, Grant WE, Teel PD, Lohmeyer KH, Pérez de León AA. Enhanced biosurveillance of high-consequence invasive pests: Southern cattle fever ticks,

Rhipicephalus (*Boophilus*) *microplus*, on livestock and wildlife. Parasites & Vectors. 2020;13:487.

3. Mount GA, Haile DG, Davey RB, Cooksey LM. Computer simulation of boophilus cattle tick (Acari: Ixodidae) POPULATION DYNAMICS. Journal of Medical Entomology. 1991;28 2:223-40; doi: 10.1093/jmedent/28.2.223. https://doi.org/10.1093/jmedent/28.2.223.
4. Cooksey LM, Davey RB, Ahrens EH, George JE. Suitability of White-Tailed Deer as Hosts for Cattle Fever Ticks (Acari: Ixodidae). Journal of Medical Entomology. 1989;26 3:155-8; doi: 10.1093/jmedent/26.3.155. https://doi.org/10.1093/jmedent/26.3.155.
